# Flake Electrical Conductivity of Few-Layer Graphene

**DOI:** 10.1155/2014/581478

**Published:** 2014-01-16

**Authors:** Hamze Mousavi, Jabbar Khodadadi

**Affiliations:** ^1^Department of Physics, Razi University, Kermanshah, Iran; ^2^Nanoscience and Nanotechnology Research Center, Razi University, Kermanshah, Iran

## Abstract

The Kubo formula for the electrical conductivity of per stratum of few-layer graphene, up to five, is analytically calculated in both simple and Bernal structures within the tight-binding Hamiltonian model and Green's function technique, compared with the single-layer one. The results show that, by increasing the layers of the graphene as well as the interlayer hopping of the nonhybridized *p*_*z*_ orbitals, this conductivity decreases. Although the change in its magnitude varies less as the layer number increases to beyond two,distinguishably, at low temperatures, it exhibits a small deviation from linear behavior. Moreover, the simple bilayer graphene represents more conductivity with respect to the Bernal case.

Graphene is an atom thick allotrope of carbon in two-dimensional (2D) hexagonal honeycomb lattice [1]. Electrons in a single-layer graphene exhibit a characteristic linear dispersion relation between energy and momentum near the *K* point of the first Brillouin zone (FBZ) [2–4]. The overall electronic structure changes sensitively with increasing crystallographic stacking sequence. The sequence of graphene sheets brings about the various 3D graphite crystals [5–8], that is, *AA*-stacked bilayer graphene (hexagonal simple bilayer graphene), *AB*-stacked bilayer graphene (Bernal bilayer graphene), and *AB* *C*-stacked trilayer graphene (rhombohedral trilayer graphene). The interlayer interactions, due to the weak overlap of the nonhybridized *p*_*z*_ orbitals, result in the anisotropic band structure along the stacking direction. Some theoretical studies [9–12] have predicted in two or more layers of graphene that the linearly dispersing bands are either replaced or augmented by split hyperbolic bands. Experimental investigations have also been considered to single- and bilayer graphene [13–15]. In a single-layer graphene transistor, the current is modulated by a gate voltage but it cannot be switched off due to lack of a band gap in the energy dispersion. Bilayer graphene is the only known semiconductor with a gate tuneable band gap [16]. Opposed to the case of single- and bilayer, the trilayer material is a semimetal with a gate tuneable band overlap between the conduction and the valence bands [16]. The variety of electronic properties found in different few-layer graphene (FLG) is the true strength of these newly discovered materials.

In this study, the electrical conductivity (EC) of FLG in {*AA*, *AA* *A*, *AA* *AA*, *AA* *AA* *A*, *AB*, *AB* *A*, *AB* *AB*, *AB* *AB* *A*} structures is investigated within the tight-binding (TB) Hamiltonian model and Green's function method. Using band representation of Green's function, we calculate the EC of the systems by Kubo formula [17–19]. Then, the temperature-dependent EC of a monolayer graphene will be compared to that for per sheet of these systems, hereinafter mentioned as ‘‘flake EC” (FEC).

In second quantization form, the Hamiltonian of the TB model for FLG lattice reads as follows [20]:

(1) $$\begin{array}{c} ℋ=-\underset{\alpha ,\beta \;}{\sum }\overset{N_{c}}{\underset{i,j=1\;}{\sum }}\overset{N_{p}}{\underset{p,q=1}{\sum }}t_{ipjq}^{\alpha \beta }c_{ip}^{\alpha †}c_{jq}^{\beta }, \end{array}$$

where *α* and *β* refer to the *A* or *B* subsites inside the Bravais lattice unit cells (Figure 1) in each plane of the system, *i* and *j* denote the position of the Bravais unit cell in the lattice, *p* and *q* describe plane's indexes, *N*_*c*_ shows the number of the Bravais lattice unit cell, *N*_*p*_ implies the number of the layers, *t*_*ipjq*_^*αβ*^ presents the amplitude for a *π* electron to hop from the subsite *α* of the Bravais lattice site *i* in plane *p* to the subsite *β* of the nearest-neighbor (NN) site *j* in plane *q*, and *c*_*ip*_^*α*†^  (*c*_*jq*_^*β*^) displays the creation (annihilation) operator of an electron on subsite *α*  (*β*) of the Bravais lattice site *i* (*j*) in plane *p*  (*q*). In our calculations, we take the chemical potential equal to zero corresponding to the contribution of one electron per *p*_*z*_ orbital in the system. We note that the on-site energy of the carbon atoms has been fitted as zero. Besides, such units are utilized that *ℏ* = *k*_*B*_ = *m*_*e*_ = *e* = 1.

We study the Hamiltonian Equation (1) by Green's function approach. Since each Bravais lattice unit cell of the graphene sheet includes *N*_*a*_ = 2 atoms, the Hamiltonian of the trilayers graphene, *N*_*p*_ = 3, as a typical case, would be introduced by a 6 × 6 matrix with the following basis kets of the Hilbert space,

(2) $$\begin{array}{c} \left\{|\Phi_{ip}^{\alpha }\rangle \right\}=\left\{|\Phi_{i1}^{A}\rangle ,|\Phi_{i2}^{A}\rangle ,|\Phi_{i3}^{A}\rangle ,|\Phi_{i1}^{B}\rangle ,|\Phi_{i2}^{B}\rangle ,|\Phi_{i3}^{B}\rangle \right\}, \end{array}$$

so the trilayer Green function could be represented as

(3) $$\begin{array}{c} G\left(i,j;\tau \right)=\left(\begin{array}{cccccc} G_{11}^{AA} & G_{12}^{AA} & G_{13}^{AA} & G_{11}^{AB} & G_{12}^{AB} & G_{13}^{AB}\\ G_{21}^{AA} & G_{22}^{AA} & G_{23}^{AA} & G_{21}^{AB} & G_{22}^{AB} & G_{23}^{AB}\\ G_{31}^{AA} & G_{32}^{AA} & G_{33}^{AA} & G_{31}^{AB} & G_{32}^{AB} & G_{33}^{AB}\\ G_{11}^{BA} & G_{12}^{BA} & G_{13}^{BA} & G_{11}^{BB} & G_{12}^{BB} & G_{13}^{BB}\\ G_{21}^{BA} & G_{22}^{BA} & G_{23}^{BA} & G_{21}^{BB} & G_{22}^{BB} & G_{23}^{BB}\\ G_{31}^{BA} & G_{32}^{BA} & G_{33}^{BA} & G_{31}^{BB} & G_{32}^{BB} & G_{33}^{BB} \end{array}\right), \end{array}$$

with *G*_*pq*_^*αβ*^(*i*, *j*; *τ*) ≡ *G*_*pq*_^*αβ*^ = −〈*𝒯c*_*ip*_^*α*^(*τ*)*c*_*jq*_^*β*†^(0)〉, in which *τ* = *it* remarks imaginary time and *𝒯* hints the time ordering operator. Here, 〈⋯〉 exhibits ensemble averaging on the ground state of the system. Using Green's function formalism for the Hamiltonian in (1), the equation of motion for electrons in *AA* *A* structure can be written as

(4) $$\begin{array}{c} \underset{\ell }{\sum }\left(\begin{array}{cccccc} E & t_{\langle i1\ell 2\rangle }^{AA} & 0 & t_{\langle i1\ell 1\rangle }^{AB} & 0 & 0\\ t_{\langle i2\ell 1\rangle }^{AA} & E & t_{\langle i2\ell 3\rangle }^{AA} & 0 & t_{\langle i2\ell 2\rangle }^{AB} & 0\\ 0 & t_{\langle i3\ell 2\rangle }^{AA} & E & 0 & 0 & t_{\langle i3\ell 3\rangle }^{AB}\\ t_{\langle i1\ell 1\rangle }^{BA} & 0 & 0 & E & t_{\langle i1\ell 2\rangle }^{BB} & 0\\ 0 & t_{\langle i2\ell 2\rangle }^{BA} & 0 & t_{\langle i2\ell 1\rangle }^{BB} & E & t_{\langle i2\ell 3\rangle }^{BB}\\ 0 & 0 & t_{\langle i3\ell 3\rangle }^{BA} & 0 & t_{\langle i3\ell 2\rangle }^{BB} & E \end{array}\right)G\left(\ell ,j;E\right)=I\delta_{ij}, \end{array}$$

and that for *AB* *A* case is given by

(5) $$\begin{array}{c} \underset{\ell }{\sum }\left(\begin{array}{cccccc} E & 0 & 0 & t_{\langle i1\ell 1\rangle }^{AB} & 0 & 0\\ 0 & E & 0 & t_{\langle i2\ell 1\rangle }^{AB} & t_{\langle i2\ell 2\rangle }^{AB} & t_{\langle i2\ell 3\rangle }^{AB}\\ 0 & 0 & E & 0 & 0 & t_{\langle i3\ell 3\rangle }^{AB}\\ t_{\langle i1\ell 1\rangle }^{BA} & t_{\langle i1\ell 2\rangle }^{BA} & 0 & E & 0 & 0\\ 0 & t_{\langle i2\ell 2\rangle }^{BA} & 0 & 0 & E & 0\\ 0 & t_{\langle i3\ell 2\rangle }^{BA} & t_{\langle i3\ell 3\rangle }^{BA} & 0 & 0 & E \end{array}\right)G\left(\ell ,j;E\right)=I\delta_{ij}, \end{array}$$

where *E* = *ℰ* + *ı*0^+^, the index 〈⋯〉 shows NN sites, **I** plays the role of a 6 × 6 unit matrix, and *δ*_*ij*_ notifies the Kronecker symbol. The **k**-space Fourier transformation of (4) and (5) leads to the following relations:

(6) $$\begin{array}{c} G\left(k;E\right)={\left(\begin{array}{cccccc} E & t_{⊥} & 0 & \epsilon_{k} & 0 & 0\\ t_{⊥} & E & t_{⊥} & 0 & \epsilon_{k} & 0\\ 0 & t_{⊥} & E & 0 & 0 & \epsilon_{k}\\ \epsilon_{k}^{*} & 0 & 0 & E & t_{⊥} & 0\\ 0 & \epsilon_{k}^{*} & 0 & t_{⊥} & E & t_{⊥}\\ 0 & 0 & \epsilon_{k}^{*} & 0 & t_{⊥} & E \end{array}\right)}^{-1},\;\;\\ G\left(k;E\right)={\left(\begin{array}{cccccc} E & 0 & 0 & \epsilon_{k} & 0 & 0\\ 0 & E & 0 & t_{⊥} & \epsilon_{k} & t_{⊥}\\ 0 & 0 & E & 0 & 0 & \epsilon_{k}\\ \epsilon_{k}^{*} & t_{⊥} & 0 & E & 0 & 0\\ 0 & \epsilon_{k}^{*} & 0 & 0 & E & 0\\ 0 & t_{⊥} & \epsilon_{k}^{*} & 0 & 0 & E \end{array}\right)}^{-1}, \end{array}$$

in which *ϵ*_**k**_ is defined as

(7) $$\begin{array}{c} \epsilon_{k}=t_{||}\left[1+2\exp\left(ı\sqrt{3}k_{x}\frac{a}{2}\right)\cos\left(k_{y}\frac{a}{2}\right)\right], \end{array}$$

where  **k** = (*k*_*x*_, *k*_*y*_) points a 2D wave vector in the FBZ, $a=|a_{1}|=|a_{2}|=\sqrt{3}a_{0}$, in which *a*_0_ displays interatomic distance, and {**a**_1_, **a**_2_} perform as primitive vectors (Figure 1). In (6)-(7), the intraplane hopping to the NN sites and the interplane ones are denoted by *t*_||_ and *t*_⊥_, respectively (Figure 2).

Our starting point for the EC tensor is the well-known Kubo formula [17–19],

(8) $$\begin{array}{c} \sigma_{\mu \nu }\left(T\right)=\overset{+\infty }{\underset{-\infty }{\int }}dℰ\left[-\partial_{ℰ}f\left(ℰ,T\right)\right]\xi_{\mu \nu }\left(ℰ\right), \end{array}$$

where {*μ*, *ν*} indicate Cartesian components, *T* shows temperature, and *f*(*ℰ*, *T*) refers to Fermi-Dirac distribution function, *f*(*ℰ*, *T*) = [1 + exp(*ℰ*/*T*)]^−1^. In band representation, energy-dependent EC, *ξ*_*μν*_(*ℰ*), is defined as

(9) $$\begin{array}{c} \xi_{\mu \nu }\left(ℰ\right)=\frac{1}{\pi N_{b}N_{c}\Omega_{c}}\overset{\text{FBZ}}{\underset{k}{\sum }}\overset{N_{b}}{\underset{b=1}{\sum }}v_{\mu }^{\left(b\right)}\left(k\right)v_{\nu }^{\left(b\right)}\left(k\right){\left[ℑG^{\left(b\right)}(k;E)\right]}^{2}, \end{array}$$

in which *b* serves as band index, *N*_*b*_ = *N*_*a*_ *N*_*p*_ equals the number of the bands in the system, *v*_*μ*_^(*b*)^(**k**) = ∂_*k*_*μ*__ *ℰ*_0_^(*b*)^(**k**) describes a *b* band Cartesian component of the velocity operator, and *ℰ*_0_^(*b*)^(**k**)'s express eigenvalues of the Hamiltonian of the system. We point out that, in band representation, the Hamiltonian of the system has a diagonal form, so (6) get the shape as

(10) $$\begin{array}{c} G\left(k;E\right)={\left(\begin{array}{cccccc} \zeta^{\left(1\right)}\left(k\right) & 0 & 0 & 0 & 0 & 0\\ 0 & \zeta^{\left(2\right)}\left(k\right) & 0 & 0 & 0 & 0\\ 0 & 0 & \zeta^{\left(3\right)}\left(k\right) & 0 & 0 & 0\\ 0 & 0 & 0 & \zeta^{\left(4\right)}\left(k\right) & 0 & 0\\ 0 & 0 & 0 & 0 & \zeta^{\left(5\right)}\left(k\right) & 0\\ 0 & 0 & 0 & 0 & 0 & \zeta^{\left(6\right)}\left(k\right) \end{array}\right)}^{-1}, \end{array}$$

where *ζ* ^(*b*)^(**k**) = *E* − *ℰ*_0_^(*b*)^(**k**). For monolayer graphene, *N*_*p*_ = 1, *ℰ*_0_^(*b*)^(**k**)'s are calculated as

(11) $$\begin{array}{c} {ℰ}_{0}^{\left(1\right)}\left(k\right)=-{ℰ}_{0}^{\left(2\right)}\left(k\right)=\left|\epsilon_{k}\right|, \end{array}$$

so that

(12) $$\begin{array}{c} \left|\epsilon_{k}\right|=t_{||}{\left\{1+4\left[\cos\left(\sqrt{3}k_{x}\frac{a}{2}\right)+\cos\left(k_{y}\frac{a}{2}\right)\right]\cos\left(k_{y}\frac{a}{2}\right)\right\}}^{1/2}. \end{array}$$

For FEG, up to five layers, the eigenvalues could also be analytically found. Using (10), *G* ^(*b*)^(**k**; *E*) turns out to be

(13) $$\begin{array}{c} G^{\left(b\right)}\left(k;E\right)=\frac{1}{E-{ℰ}_{0}^{\left(b\right)}\left(k\right)}. \end{array}$$

Moreover, the velocity operator could be represented by

(14) $$\begin{array}{c} v_{\mu }\left(k\right)=\left(\begin{array}{cccccc} v_{\mu }^{\left(1\right)}\left(k\right) & 0 & 0 & 0 & 0 & 0\\ 0 & v_{\mu }^{\left(2\right)}\left(k\right) & 0 & 0 & 0 & 0\\ 0 & 0 & v_{\mu }^{\left(3\right)}\left(k\right) & 0 & 0 & 0\\ 0 & 0 & 0 & v_{\mu }^{\left(4\right)}\left(k\right) & 0 & 0\\ 0 & 0 & 0 & 0 & v_{\mu }^{\left(5\right)}\left(k\right) & 0\\ 0 & 0 & 0 & 0 & 0 & v_{\mu }^{\left(6\right)}\left(k\right) \end{array}\right). \end{array}$$

From (9), (10), and (13) and definition of velocity, the *x*-component of the energy-dependent EC, *ξ*_*xx*_(*ℰ*), of the simple structures (*N*_*p*_ = 1,2, 3,4, 5) can be written as

(15) ξxx(ℰ)=ξ0∑kFBZ{[sin2(3kx(a/2))cos2(ky(a/2))|ϵk|2]×∑b=1Nb[ℑ(1E−ℰ0(b)(k))]2},

while that of the Bernal structure with *N*_*p*_ = 2 is determined by

(16) ξxx(ℰ)=ξ0∑kFBZ{[sin2(3kx(a/2))cos2(ky(a/2))|ϵk|2+(t⊥/2)2]×∑b=1Nb[ℑ(1E−ℰ0(b)(k))]2};

for *N*_*p*_ = 3, the result is

(17) ξxx(ℰ) =ξ0∑kFBZsin2(3kxa2)cos2(kya2)     ×{(1|ϵk|2+(2(t⊥/2))2)×∑b=14′[ℑ(1E−ℰ0(b)(k))]2+(1|ϵk|2)∑b=56′[ℑ(1E−ℰ0(b)(k))]2}.

When *N*_*p*_ = 4, it is found that

(18) ξxx(ℰ) =ξ0∑kFBZsin2(3kxa2)cos2(kya2)    ×{[1|ϵk|2+((3+5)/2)(t⊥/2)2]×∑b=14′[ℑ(1E−ℰ0(b)(k))]2+[1|ϵk|2+((3−5)/2)(t⊥/2)2]×∑b=58′[ℑ(1E−ℰ0(b)(k))]2},

and *N*_*p*_ = 5 leads to

(19) ξxx(ℰ) =ξ0∑kFBZsin2(3kxa2)cos2(kya2)    ×{[1|ϵk|2+(3(t⊥/2))2]×∑b=14′[ℑ(1E−ℰ0(b)(k))]2+[1|ϵk|2+(t⊥/2)2]×∑b=58′[ℑ(1E−ℰ0(b)(k))]2+(1|ϵk|2)∑b=910′[ℑ(1E−ℰ0(b)(k))]2},

in which *ξ*_0_ = 3*a* ^2^ *t*_||_^4^/(*πN*_*b*_ *N*_*c*_ *Ω*_*c*_) and ∑′ implies sum over just some bands not all.

In summary, using Green's function method and the Kubo-Greenwood formula through the TB Hamiltonian model, the EC of FLG is analytically found for single-layer and {*AA*, *AA* *A*, *AA* *AA*, *AA* *AA* *A*} simple cases as well as {*AB*, *AB* *A*, *AB* *AB*, *AB* *AB* *A*} Bernal structures. The aim is to compare the EC of the single-layer graphene and FLG (see (8) and (11)–(19)). We set the intraplane hopping to the NN and interplane ones as *t*_||_≃2.8 eV and *t*_⊥_ = *t*_||_/7≃0.4 eV [21–23], respectively. Figures (3) and (4) show the results. In Figure 3(a), the FECs of mono-layer graphene and FLG in simple structure are plotted, while, in Figure 3(b), those of the Bernal cases are classified. The latter are investigated and summarized in Figure 3(c) for *N*_*p*_ = 2 and different values of interplane term; that is, *t*_⊥_ = *t*_||_/14, *t*_||_/7, *t*_||_/4.67, and *t*_||_/3.5. Also, *σ*_*xx*_(*T*) is illustrated for simple and Bernal bilayer graphene in Figure 4. We mention that, for a monolayer graphene, *Ω*_*c*_ is just the area of the graphene unit cell. For FLG, the FEC is of more interest, so *Ω*_*c*_ will be the multiplication of the single-layer area by the layer number *N*_*p*_. So, in (15)–(19), *Ω*_*c*_ = *N*_*p*_, if we set the area of the graphene unit cell equal to unity. Numerically, we insert *a*_0_ = 1 and *t*_||_ = 0.28.

In Figures 3(a) and 3(b), the temperature-dependent FECs of mono-layer graphene and FLG are compared in simple and Bernal form as well. It is shown that *σ*_*xx*_(*T*) decreases with increasing the number of layers from *N*_*p*_ = 1 to 5. This behavior could be justified by overlapping of the nonhybridized *p*_*z*_ orbitals perpendicular to the sheets, so that these interlayer interactions will generate new channels of electron motion with respect to those of the isolated single layer, but perpendicular to them. Reasonably, these vertical detour ways can distract a fraction of the electrons' motivation from horizontal traces parallel to the layers, towards the vertical tracks. In other words, overlapped nonhybridized *p*_*z*_ orbitals lead to a partial deviation of the electrons' mobility from the planes on behalf of the normal directions. Consequently, these interlayer possibilities of movement result in a reduction of the intralayer displacements, whereby the system exhibits a decay in the FEC. This phenomenon gets more remarkable with respect to the monolayer case by increasing the number of the layers, easily conceivable from Figures 3(a) and 3(b) and in agreement with the last explanation, because adding more layers provides more distracting paths for moving electrons, so the plane components get lesser. On the other hand, the changing in the FEC could also be a result of significant variations in the low energy band structures (linear to quadratic dispersion) and the corresponding density of states (DOS). As the layer number increases to five, the variation of the low energy band structure is much less dramatic than the transition from one to two [24]. So the variation in the magnitude of the FEC changes less as the layer number increases to beyond two. Considerably, this change in dispersion provides a small deviation in the linear behavior of the FEC at low temperatures. This is more notable for the simple case whose dispersion bears more variations around the Fermi level.

It is also resulted that the FEC depends on the amount of the interplane hopping integrals. In Figure 3(c), the temperature-dependent FEC of bilayer Bernal structure is plotted for four values of *t*_⊥_. Obviously, the more *t*_⊥_ increases, the more *σ*_*xx*_(*T*) decreases. This could be interpreted that the procedure of increasing the interlayer hopping transforms the interlayer interactions towards the limit of somehow covalence-like bonds, which resemble the carbonic system as an insulator with four strong bonds and consequently weak EC.

We have also compared *σ*_*xx*_(*T*) of bilayer both simple and Bernal graphene in Figure 4. It is known that a finite DOS at zero energy appears in the simple case [25] in contrast to the single layer whose DOS vanishes. Therefore, because of appearing allowed states close to Fermi energy, the temperature-dependent FEC of the simple case is more that of the Bernal one.

Totally, it is concluded that the FEC decreases by increasing the layers of the graphene due to overlapping of the nonhybridized *p*_*z*_ orbitals perpendicular to the sheets. But, the variation in the magnitude of the FEC varies less as the layer number increases to beyond two as a result of changes in the low energy band structures. Besides, a deviation from the linear behavior of the FEC is observable at low temperatures originated from changes in relevant dispersion, especially for the simple case. It is found that more increase in interplane term causes more decrease in the temperature-dependent FEC because of transforming the interlayer interactions towards the limit of covalence-like bonds. Finally, it is resulted that the FEC of simple structure is more than that of Bernal one.

## Figures and Tables

**Figure 1:**
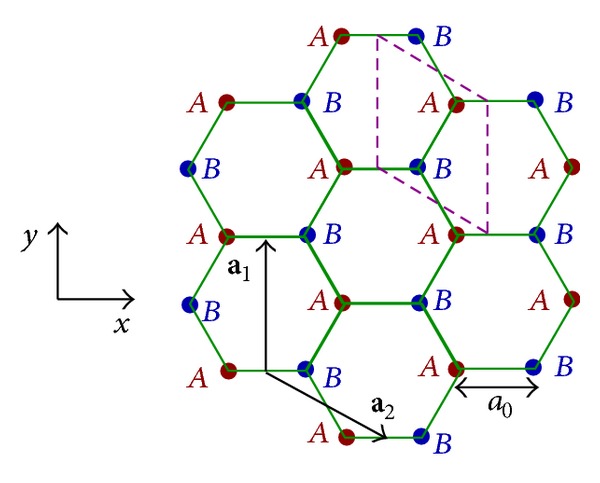
Geometry of monolayer graphene in *xy* plane. The dashed lines illustrate the Bravais lattice unit cell. Each cell includes *N*_*a*_ = 2 atoms, which are shown by *A* and *B*. The primitive vectors are denoted by **a**_1_ and **a**_2_ and *a*_0_ implies the interatomic distance.

**Figure 2:**
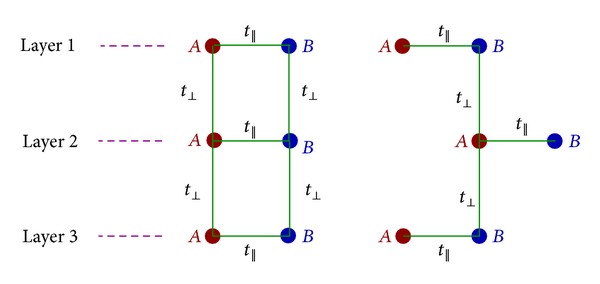
Schematic presentation of intra- (*t*_||_) and interplane (*t*_⊥_) hopping to the NN sites in trilayer graphene for simple case (left) and Bernal one (right).

**Figure 3:**
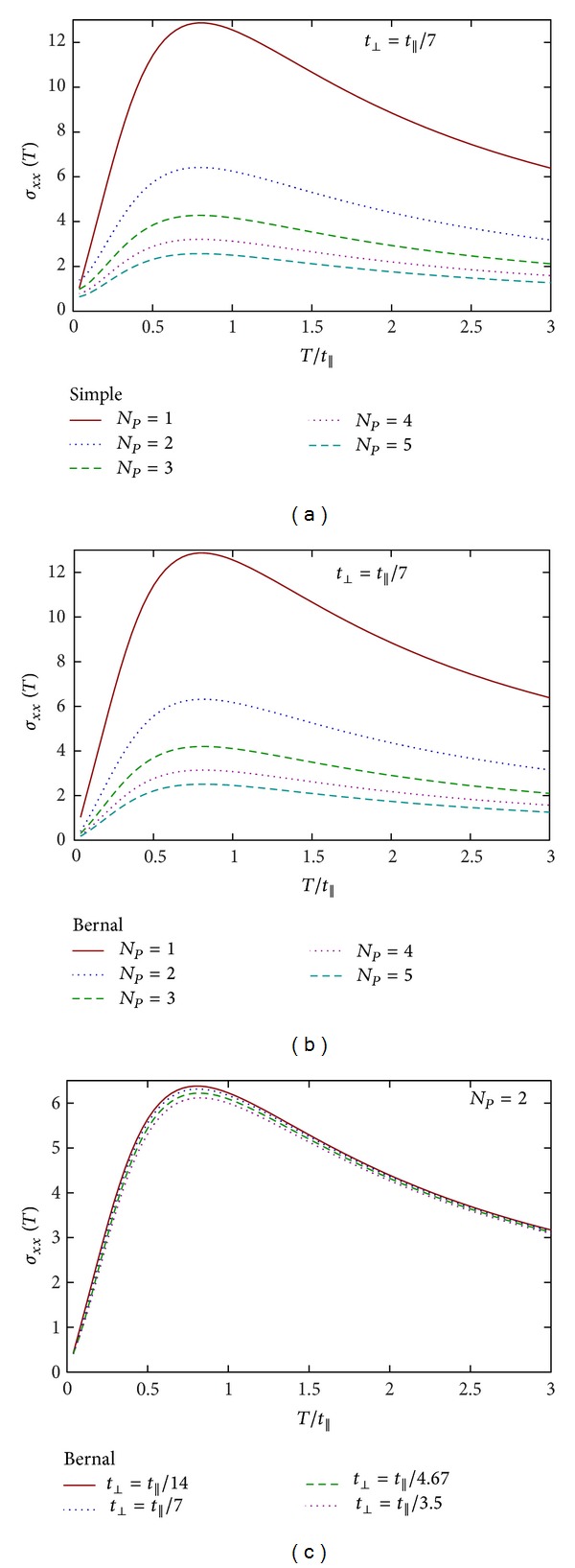
The FEC of mono-, bi-, tri-, tetra-, and pentalayer graphene plane for simple structure (a) and Bernal case (b). In (a) and (b), the interplane hopping term is chosen to be *t*_⊥_ = *t*_||_/7. (c) shows the FEC of the bilayer Bernal graphene for four values of interplane hopping term, *t*_⊥_ = *t*_||_/14, *t*_||_/7, *t*_||_/4.67, and *t*_||_/3.5.

**Figure 4:**
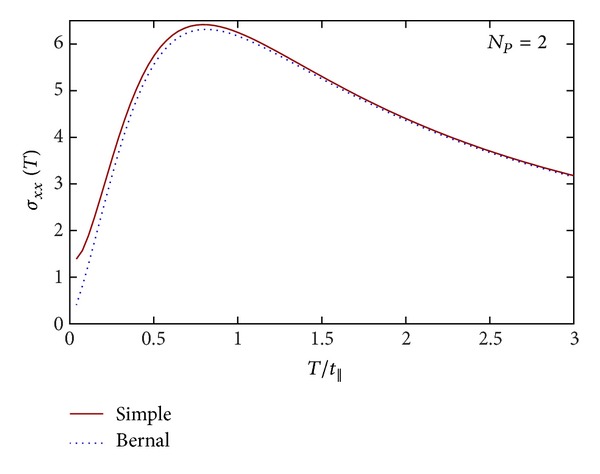
Comparison of the FEC of the bilayer graphene for simple and Bernal cases. The interplane hopping term is *t*_⊥_ = *t*_||_/7.
